# Intranasal Transplantation of Human Neural Stem Cells Ameliorates Alzheimer's Disease-Like Pathology in a Mouse Model

**DOI:** 10.3389/fnagi.2021.650103

**Published:** 2021-03-10

**Authors:** Mei-Hong Lu, Wen-Li Ji, Hong Chen, Yan-Yun Sun, Xiu-Yun Zhao, Fen Wang, Yi Shi, Yan-Ning Hu, Bo-Xiang Liu, Jing-wen Wu, De-En Xu, Jia-Wei Zheng, Chun-Feng Liu, Quan-Hong Ma

**Affiliations:** ^1^Jiangsu Key Laboratory of Neuropsychiatric Diseases, Institute of Neuroscience, Soochow University, Suzhou, China; ^2^School of Medical Technology, Chengdu University of Traditional Chinese Medicine, Chengdu, China; ^3^Department of Functional Neurology, Shanghai East Hospital, Tongji University, Shanghai, China; ^4^Department of Neurology, Wuxi No. 2 People's Hospital, Wuxi, China; ^5^Angecon Biotechnology Co., Ltd., Shanghai, China

**Keywords:** neural stem cell, transplantation, intranasal, neurological disorder, Alzheimer's disease, cell therapy

## Abstract

Alzheimer's disease (AD) is a neurodegenerative disorder characterized by memory impairments, which has no effective therapy. Stem cell transplantation shows great potential in the therapy of various disease. However, the application of stem cell therapy in neurological disorders, especially the ones with a long-term disease course such as AD, is limited by the delivery approach due to the presence of the brain blood barrier. So far, the most commonly used delivery approach in the therapy of neurological disorders with stem cells in preclinical and clinical studies are intracranial injection and intrathecal injection, both of which are invasive. In the present study, we use repetitive intranasal delivery of human neural stem cells (hNSCs) to the brains of APP/PS1 transgenic mice to investigate the effect of hNSCs on the pathology of AD. The results indicate that the intranasally transplanted hNSCs survive and exhibit extensive migration and higher neuronal differentiation, with a relatively limited glial differentiation. A proportion of intranasally transplanted hNSCs differentiate to cholinergic neurons, which rescue cholinergic dysfunction in APP/PS1 mice. In addition, intranasal transplantation of hNSCs attenuates β-amyloid accumulation by upregulating the expression of β-amyloid degrading enzymes, insulin-degrading enzymes, and neprilysin. Moreover, intranasal transplantation of hNSCs ameliorates other AD-like pathology including neuroinflammation, cholinergic dysfunction, and pericytic and synaptic loss, while enhancing adult hippocampal neurogenesis, eventually rescuing the cognitive deficits of APP/PS1 transgenic mice. Thus, our findings highlight that intranasal transplantation of hNSCs benefits cognition through multiple mechanisms, and exhibit the great potential of intranasal administration of stem cells as a non-invasive therapeutic strategy for AD.

## Introduction

Alzheimer's disease (AD) is a common progressive neurodegenerative disease, which is clinically manifested as memory loss, speaking and problem-solving difficulties, and other cognitive impairments. The extracellular accumulation of β-amyloid (Aβ) plaques and intracellular neurofibrillary tangles are the two main pathological features (Hampel et al., 2015). Especially, Aβ is believed to be the instigator of the whole AD event, which leads to neurofibrillary tangles, synapse loss, neuroinflammation, neuronal death, and cognitive deficits (Long and Holtzman, 2019). Unfortunately, all current clinical trials targeting Aβ, tau, and even neuroinflammation have failed so far (Long and Holtzman, 2019). Current drugs, such as acetylcholinesterase inhibitors or N-methyl-D-aspartic acid receptor antagonists, are only beneficial for AD symptoms. Last year, a new multi-targets drug, GV-971, was conditionally approved for sale in China but only used for the treatment of mild to moderate AD (Wang et al., 2019). Despite decades of efforts, there is no effective treatment to cure AD. Therefore, finding more strategies for AD therapy is of great importance.

With advances in stem cell techniques, therapies based on it have already emerged as a novel and promising therapeutic strategy for a variety of neurological disorders (Lindvall and Kokaia, 2010; Lee et al., 2015). Such therapies offer beneficial effects via multiple mechanisms, including replacement of damaged cells, neuroprotection through neurotrophic factors release, or anti-inflammatory effects (Bali et al., 2017; Duncan and Valenzuela, 2017). Neural stem cells (NSCs) are self-renewing cells that can differentiate into neurons, astrocytes, and oligodendrocytes (Teng, 2019). A number of studies have reported that the transplantation of murine NSCs improves the learning memory in an AD mice model (Zhang et al., 2014; Gu et al., 2015; Kim et al., 2015). A study indicates that human NSCs (hNSCs) also have beneficial effects in an AD model, which suggests the potential for clinical application in future (Hayashi et al., 2020). However, due to the presence of the brain blood barrier (BBB), most stem cell therapies for neurological disorders in preclinical studies and clinical trials use direct intracranial or intrathecal injection to deliver the cells directly to the brain, which hampers clinical application due to its invasion and possible side effects (Reyes et al., 2015). By contrast, in the past decade, intranasal administration has received a great deal of attention as an alternative route in the delivery of proteins, gene vectors, exosomes, and even cells to the central nervous system (CNS), which by-passes the BBB and harbors non-invasive and safe advantages (Lochhead and Thorne, 2012; Guo et al., 2019). Moreover, intranasal delivery allows for repetitive administration, which provides a great advantage for a long-term course disease such as AD. However, whether repetitive intranasal transplantation of hNSCs could improve AD cognition is still unclear. In this study, we administrated hNSCs repetitively via an intranasal delivery approach, which were derived from embryos aborted at 6–8 weeks of gestation, to the brains of APP/PS1 transgenic mice who exhibit AD-like pathology at an older age. We investigated the therapeutic potential of repetitive intranasal transplantation of hNSCs in APP/PS1 transgenic mice. We observed that intranasally transplanted hNSCs survived, migrated extensively, and differentiated into higher numbers of neurons, and relatively fewer astrocytes and oligodendrocytes in the host brain. Intranasal transplantation of hNSCs attenuated the following pathological processes in the host brains: (1) accumulation of Aβ, concomitant with increased levels of insulin-degrading enzyme (IDE) and neprilysin (NEP), the key Aβ-degraded enzymes; (2) cholinergic dysfunction with a portion of transplanted hNSCs differentiating into cholinergic neurons; (3) pericytic and synaptic loss, accompanied with elevated levels of vascular endothelial growth factor (VEGF); and (4) neuroinflammation. Moreover, intranasal transplantation of hNSCs enhances the adult hippocampal neurogenesis (AHN) of host mice. Consistent with these observations, intranasal transplantation of hNSCs rescues cognitive deficits of APP/PS1 mice. Therefore, our data indicate that repetitive intranasal transplantation of hNSCs ameliorates AD-like pathology in AD model mice. These results provide preclinical evidence that there is therapeutic potential in the intranasal transplantation of hNSCs in AD patients.

## Materials and Methods

### Antibodies

Mouse anti-Stem121 (Takara, Y40410), mouse anti-GFAP (CST, 3670S), mouse anti-Tuj1 (Sigma, T8860), rabbit anti-O4 (Millipore, MAB345), rabbit anti-NG2 (Millipore, AB5320), rabbit anti-GFAP (Wako, Z0334), Alexa Fluor® 647 mouse anti-Nestin (BD, 560341), Alexa Fluor® 488 mouse anti-Sox2 (BD, 560301), Alexa Fluor® 647 mouse IgG1, κ, Isotype (BD, 557732), Alexa Fluor® 488 mouse IgG2a, κ, Isotype (BD, 558055), rabbit anti- synaptophysin (SYN) (Abcam, ab32127), mouse anti-β-Amyloid (Biolegend, 803001), rabbit anti-NeuN (CST, 12943S), rabbit anti-Doublecorxin (DCX) (CST, 4604S), rabbit anti-Iba1 (Wako, 019-19741), rabbit anti-CHAT (Abnova, pab14536), rabbit anti-APP (Sigma, A8717), mouse anti-BACE1 (NOVUS, NBP2-37261), rabbit anti-IDE (Abcam, ab133561), rabbit anti-NEP (R&D system, MAB1126), goat anti-PDGFRβ (R&D Systems, AF1042), rabbit anti-VEGF (Abcam, ab46154), mouse anti-GAPDH (Proteintech, 60004-1-Ig), mouse anti-γ-Tubulin (Sigma, T6557), and corresponding secondary antibodies conjugated with HRP (Sigma) or Alexa fluorophore 488, 555, or 647 (Invitrogen) were used.

### Mice

APP/PS1 double transgenic mice express mutant human amyloid precursor protein (APPswe) and presenilin 1 (PS1-dE9) under the mouse prion protein promoter. These transgenic mice were purchased from Jax laboratory (stock number 004462). Male mice were divided into three groups: wild-type control mice received normal saline (WT+saline), APP/PS1 control group received normal saline (TG+saline), and APP/PS1 group received stem cells (TG+hNSCs). All animals were housed under SPF conditions. All experimental procedures were approved by the Ethics Committee of Soochow University in accordance with international laws.

### Culture and Differentiation of hNSCs

hNSCs were isolated and cultured by Angecon Biotechnology Co., Ltd (Shanghai, China). hNSCs were obtained from the hippocampus of aborted embryos at 6–8 weeks of gestational age. The hNSCs were cultured in T75 culture flasks containing DMEM/12 medium supplemented with 1% GlutaMAX (Invitrogen), 2% B27 (Invitrogen), N-Acetylcysteine (1 mM, Sigma), human recombinant epidermal growth factor (EGF, 20 ng/ml, Peprotech), basic fibroblast growth factor (bFGF, 20 ng/ml, Peprotech), and leukemia inhibitory factor (LIF, 10 ng/ml, Peprotech) in a humidified 5% CO2/95 % air incubator at 37°C. For neurospheres formation, hNSCs were cultured at a density of 1 × 10^5^ cells/ml in T75 culture flasks for 12 d.

For differentiation analysis, suspended neurospheres were digested with TrypLE and then were seeded in a poly-l-lysine-coated 24-well plate for 6 h to allow the cells to fully adhere to the bottom. Then the culture medium was replaced with a cell differentiation medium containing 2% B27, 1% N2, 1% Glutamin, and 1% sodium pyruvate for 2 weeks, and the medium was changed every 3 days during this period. Then the cells were subjected to immunofluorescence staining using specific antibodies to identify Tuj1, GFAP, and O4.

### Flow Cytometry Analysis

The cell suspension containing 2 × 10^6^ cells was harvested by centrifugation at 500 g for 3 min, and were fixed and permeabilized with 1 ml of cytofix/cytoperm (Cytofix/cytoperm kit, BD) for 10 min at 4°C. After washing twice with perm-wash (Cytofix/cytoperm kit, BD), the cells were centrifuged at 300 g for 5 min and resuspended with 150 μl of perm-wash solution. The resuspension was divided into three equal parts: the controls, the isotypes, and the experimental groups. The cells were then incubated with mouse anti-Nestin conjugated with Alexa Fluor® 647 and mouse anti-Sox2 conjugated with Alexa Fluor® 488 antibodies for 30 min in the dark, washed twice with perm-wash, and resuspended with 500 μl of PBS. The control group sample had only cells, and cells from the isotype group were incubated with the corresponding isotype antibodies instead of the specific antibodies. The data were acquired using a Beckman CytoFLEX flow cytometry system (Beckman Coulter, USA) running the CytExpert software.

### Intranasal Transplantation of hNSCs

hNSCs should meet the following criteria before transplantation. The cell survival rate is about 80% at 48 h, and the endotoxin is not higher than 0.5 EU/ml. After the mice were anesthetized with isoflurane in the induction chamber, a mouse-compatible mask that can be placed around the animal's nose and mouth to maintain constant anesthesia was used. The nasal cavity of mice was treated with 4 μl of 100U/10 μl of hyaluronidase (sigma, catalog number: H3884) for half an hour to increase the permeability of the nasal sieve before stem cell transplant. During the administration process, the mouse was kept supine, and the cell suspension was slowly injected into the nasal cavity with a thin and soft tube of 0.5–0.7 cm long connected to a micro-syringe, and the mouse was kept supine for 5 min. Each mouse was received 8 μl (1 × 10^6^, 4 μl/side) of hNSCs or saline on both sides. The mice received hNSCs at 3.5 months old, once a week for 4 consecutive weeks.

All experimental animals were given an intraperitoneal injection of the immunosuppressant cyclosporine (Shandizin, Novartis, 5 ml/250 g) at a dose of 10 mg/kg for 1 week starting 2 days before the nasal transplantation. After that, they were administered cyclosporine orally at a dose of 24 mg/kg/day until the mice were sacrificed for analysis.

### Behavioral Tests

New object recognition was performed when the animals were 6.5 months old (3 months after the first administration of hNSCs or saline). The test was carried out in a 40 × 40 × 40 cm box with a camera on the top. First, the mice were put into the box for a 10-min adaptation for 3 consecutive days. Before starting the next mouse, the box was cleaned with 75% alcohol to remove odors. On the fourth day, two identical objects (A) were put on the diagonal of the box, 5 cm away from both walls. And the mouse was allowed to explore for 10 min. After 90 min, one of the A objects was replaced with a B object. The recognition index of mice was defined as the ratio of time spent on object B to the total time spent on both object A and B. Anymaze software and the one-way ANOVA statistical method were used to collect and analyze data, respectively.

The Morris water maze test were performed after NOR. The mice were placed in a large circular pool of 120 cm in diameter, surrounded by blue curtains, and covered with hanging objects of different shapes and colors. The pool was filled with opaque water and an invisible platform was submerged just below the water surface in the center of one quadrant. Animals were trained to swim in the pool and find hidden target platforms in trails four times per day for 6 consecutive days. In each trial, mice were placed in the water in a semi-random manner from four starting points (North, East, South, and West) and the test lasted for 60 s or until the mouse found the platform. On the 7th day, the platform was removed, the animals were allowed to probe the test area. A reversal test was performed with the same protocol of four trials a day for 2 days 2 weeks later. In the reversal test, the platform was relocated to the center of the opposite quadrant against the original one. The data were collected with the Anymaze software, and the escape latency and swimming speed of each group of mice were analyzed.

### Enzyme Linked Immunosorbent Assay (ELISA)

The amounts of soluble and insoluble Aβ40 and Aβ42 in the cortex and hippocampus were detected by ELISA. Briefly, mouse hemispheres were rinsed in pre-cooled PBS, and the hippocampus and cortex were separated using forceps and scissors. Then, tissues were homogenized in TBS (25 mM of Tris, 0.15M of NaCl, pH 7.2) on ice for 10 min (400 μ1 of TBS/100 mg of tissue) and centrifuged at 35,000 g at 4°C for 30 min. After collecting the supernatant (soluble fraction), the debris were resuspended and lysated with an equal volume of 5M guanidine hydrochloride for 3 h at room temperature, and centrifuged at 13,200 g for 15 min at 4°C. The collected supernatant was the insoluble fraction. Then Aβ40, Aβ42, BDNF, NGF, and NTF3 levels were detected by the ELISA kits in accordance with the ELISA instructions, respectively.

### Immunofluorescence Staining

After anesthetizing with chloral hydrate, the mice were decapitated and their brains were taken. After washing in pre-cooled PBS, the brain was fixed in 4% paraformaldehyde overnight, and then dehydrated with 15 and 30% sucrose, respectively. Then, 20 μm thick slices were made with Cryostat using OCT embedding medium. For immunofluorescence staining, the brain slices or fixed hNSCs with 4% PFA were washed with PBS three times and blocked with 10% FBS in 0.3% TritonX-100 at room temperature for 1 h. After blocking, the slices were incubated with primary antibody at 4°C overnight and the corresponding secondary antibody at room temperature for 1 h. The slides were mounted with mounting medium with DAPI and images were captured under a microscope.

### Western Blotting

Western blot analysis was carried out according to the methods described previously (Lu et al., 2019). Briefly, the brain tissue samples were homogenized with brain lysis buffer [50 mM of Tris-HCl, pH 7.5, 5 mM of EDTA, 1% Triton X-100, and protease inhibitor (Roche Diagnostics)] and centrifuged at 132,000 g for 20 min at 4°C. The supernatants were collected and subjected to SDS-PAGE electrophoresis. And the protein was transferred to the PVDF membrane followed by blocking with 5% skimmed milk dissolved in PBS-Tween 20 (0.08 M of K_2_HPO_4_, 0.02 M of KH_2_PO_4_, 0.10 M of NaCl, and 0.2% Tween20) for 1 h. The PVDF membrane was then washed three times using the PBS-Tween 20 solution and incubated with primary antibody at 4°C overnight. After washing three times, the membrane was subsequently incubated with the corresponding horseradish peroxide-conjugated secondary antibody at room temperature for 1 h. Finally, the protein signal was detected by ECL (GE Bioscience).

### Statistical Analysis

For Aβ plaques quantification, the size of the plaques was defined as the total area of plaques occupied. The mean fluorescence intensity of SYN in the hippocampal CA3 area was evaluated. For neuroinflammation and pericytes analysis, the proportion of GFAP, Iba1, or PDGFRβ positive cells-covered areas were measured. The density of DCX positive cells was defined as the total number of DCX positive cells divided by the length of the granular cell layer in the dentate gyrus (DG) region. All images were analyzed using the Image J software. All data are represented as mean ± SEM, and analyzed by SPSS 17.0. Student *t*-test was used for two group comparisons, one-way ANOVA for multiple group comparisons, and two-way ANOVA for the water maze followed by Fisher LSD test. ^*^P < 0.05; ^**^P < 0.01; ^***^P < 0.001.

## Results

### Identification of hNSCs *in vitro*

**Figure 1:**
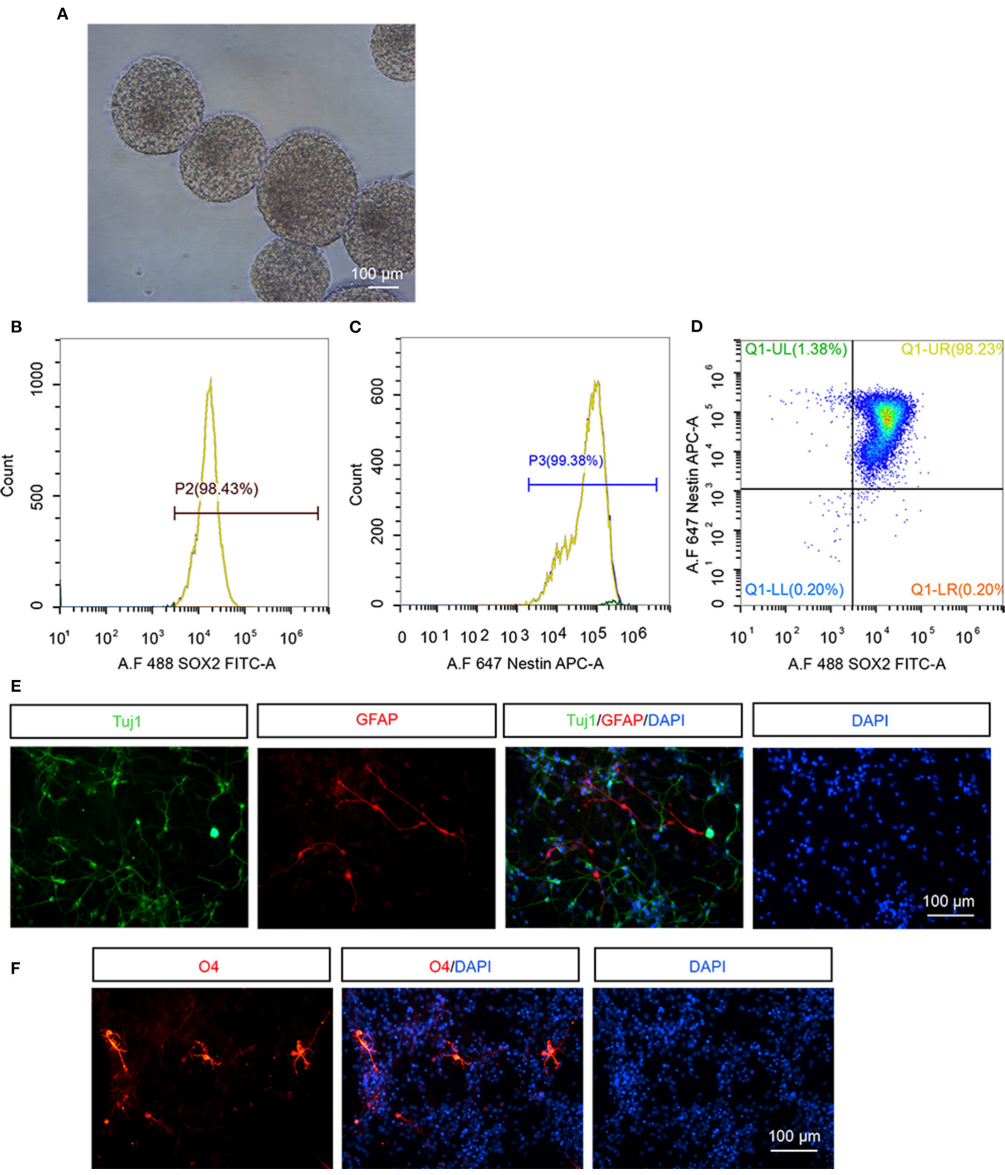
The identification of hNSCs *in vitro*. **(A)** The morphology of neurospheres of hNSCs after being cultured for 12 d. **(B–D)** Flow cytometry analysis of cultured hNSCs using antibodies against Sox2 **(B)**, Nestin **(C)**, or Sox2 and Nestin **(D)**. **(E,F)** hNSCs were cultured in a differentiation condition for 2 weeks and immunostained for Tuj1 **(E)**, GFAP **(E)**, and O4 **(F)**. Scale bar: 100 μm.

We firstly examined the property of hNSCs before transplantation. hNSCs were successfully propagated and formed neurospheres when being suspension-cultured for 12 d, indicating that hNSCs have a self-renewal ability (Figure 1A). The cultured hNSCs were further subjected to flow cytometry analysis using antibodies against stem cell specific markers, Nestin and Sox2. The results demonstrated that 98.43% of hNSCs were Sox2^+^ (Figure 1B), 99.38% were Nestin^+^ (Figure 1C), and 98.23% of hNSCs were Sox2^+^Nestin^+^ (Figure 1D), indicating the high purity of cultured hNSCs before transplantation. Upon being cultured in a differentiation condition for 2 weeks, hNSCs were immunostained for βIII-tubulin (Figure 1E, Tuj1), a marker for neurons, GFAP (Figure 1E), a marker for astrocytes, and O4 (Figure 1F), a marker for oligodendrocytes, respectively. Our data showed that hNSCs differentiated into a large number of neurons and relatively fewer astrocytes and oligodendrocytes *in vitro*. In summary, the hNSCs we isolated were of high purity and were capable of differentiation, which met the criteria for transplantation.

### Intranasally Transplanted hNSCs Survive and Migrate Extensively in Host Brain

**Figure 2:**
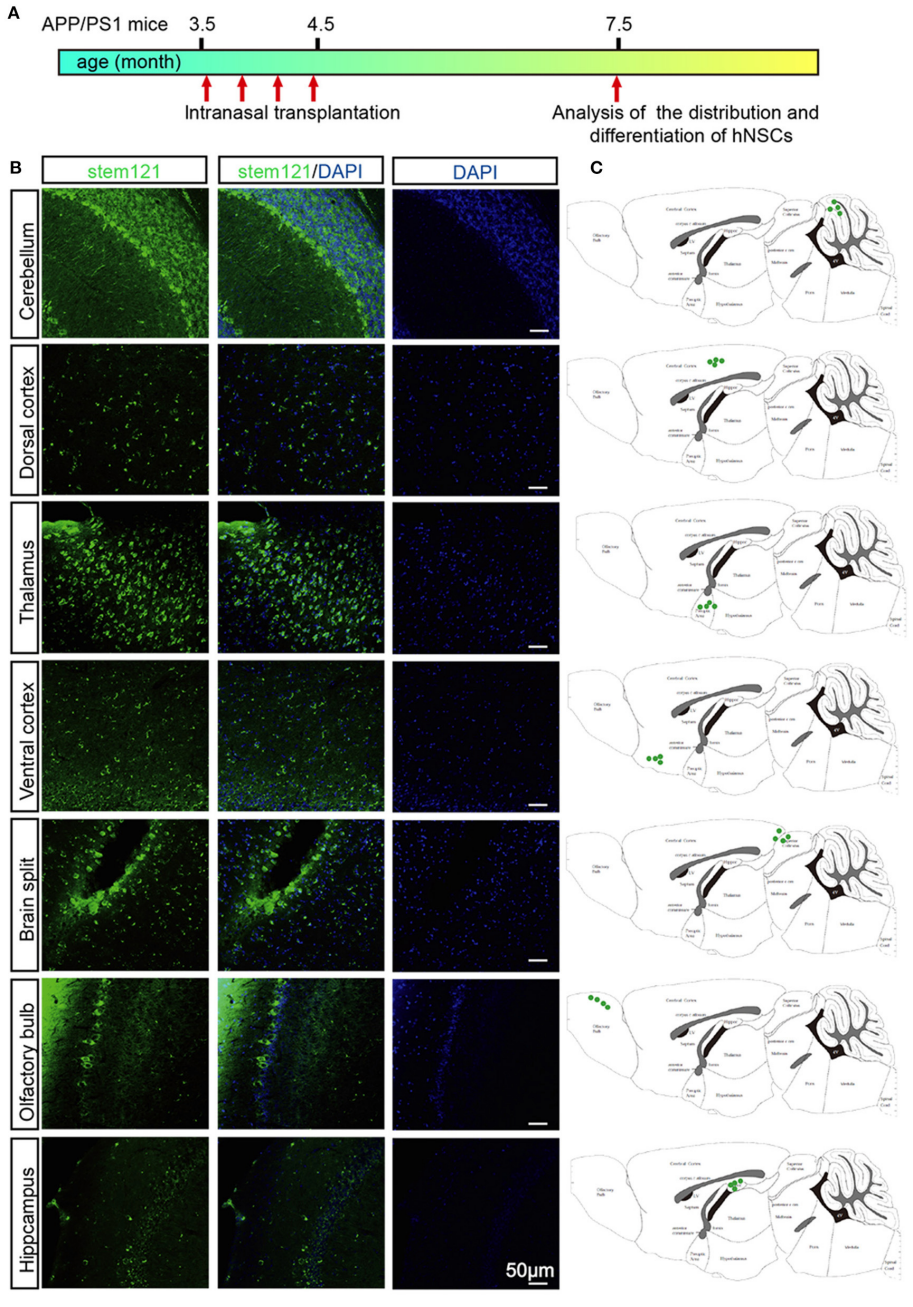
Migration of intranasally transplanted hNSCs in the brains of APP/PS1 transgenic mice. **(A)** 3.5-month-old APP/PS1 mice were transplanted with hNSCs by intranasal administration four times with a frequency of once a week, and were subjected to analysis of the distribution and differentiation of transplanted cells at 7.5 months. **(B)** The sagittal sections from three host mice were immunostained for stem121 and DAPI. **(C)** Schematic diagram drawn from three host mice showing the location of intranasal transplanted hNSCs in the host brains. Scale bars: 50 μm.

APP/PS1 transgenic mice, which express mutant APP and PS1, harbor few Aβ plaques, and show no signs of cognitive deficits at 3.5 months of age. Whereas, these transgenic mice exhibited extensive accumulation of Aβ plaques and other AD-like pathology such as neuroinflammation, loss of synapses, and cognitive deficits at 7 months old (Lu et al., 2019). APP/PS1 transgenic mice were administrated with hNSCs intranasally four times once a week at 3.5 months old and then subjected to immunohistological analysis 4 months later (Figure 2A). We first examined whether transplanted hNSCs could survive and where they would migrate to in the host brain following intranasal delivery. We labeled hNSCs with stem121, a marker specifically expressed in hNSCs, in the sagittal sections of APP/PS1 mice brains 4 months after the last intranasal delivery. We observed a large number of stem121^+^ cells in the host brains, which were distributed in the olfactory bulb, hippocampus, ventral and dorsal cortex, brain splits, thalamus, and cerebellum (Figures 2B,C). These results suggest that intranasally transplanted hNSCs survive and successfully migrate into multiple regions of the host brains.

### Intranasally Transplanted hNSCs Differentiate Into a Large Number of Neurons and Fewer Glial Cells

**Figure 3:**
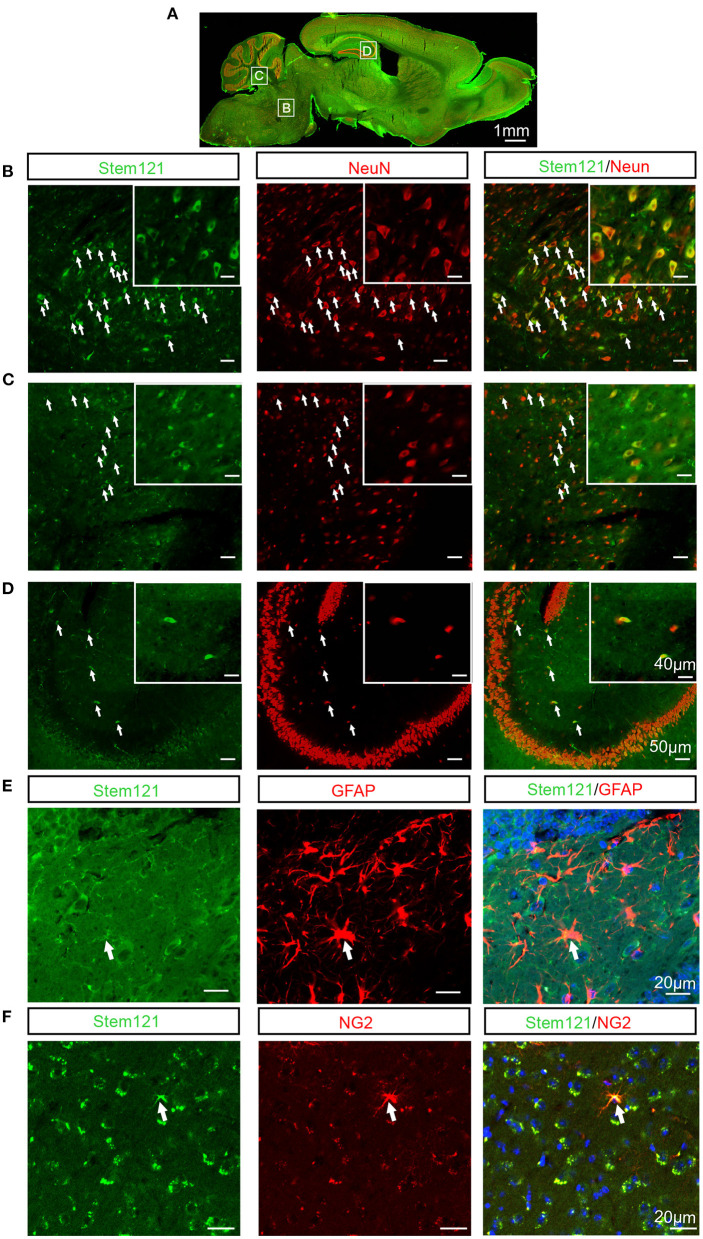
Differentiation of intranasally transplanted hNSCs in the brains of APP/PS1 transgenic mice. **(A–D)** The sagittal sections of APP/PS1 mice which were transplanted intranasally with hNSCs were coimmunostained for stem121 and NeuN. **(A)** The overall view of the fluorescence staining of the mouse sagittal section. **(B–D)** The enlarged images of the corresponding white square boxes shown in **(A)**. hNSCs pons **(B)**, cerebellum **(C)**, and hippocampus **(D)**. The images with higher magnification are shown in the inserts. The transplanted hNSCs which were differentiated into neurons are indicated by the white arrows. **(E,F)** The coronal sections of host APP/PS1 mice were coimmunostained for stem121 and GFAP **(E)** or NG2 **(F)**. The transplanted hNSCs which were differentiated into astrocytes or OPCs are indicated by the white arrows. Scale bars: 1 mm **(A)**, 50 μm in the images with lower magnification, 40 μm in the inserted images **(B–D)**, 20 μm **(E,F)**.

To test whether the intranasally transplanted hNSCs differentiated in the host brain, the sagittal sections of host APP/PS1 mice co-stained stem121 with the neuron-specific marker NeuN, the astrocyte marker GFAP, or the oligodendrocyte precursor marker NG2, respectively. The results showed that a large proportion of transplanted hNSCs differentiated into neurons (stem121^+^NeuN^+^) (Figures 3A–D). In contrast, only a small proportion of transplanted hNSCs differentiated into astrocytes (stem121^+^GFAP^+^) (Figure 3E) and oligodendrocytes (stem 121^+^NG2^+^) (Figure 3F).

**Figure 4:**
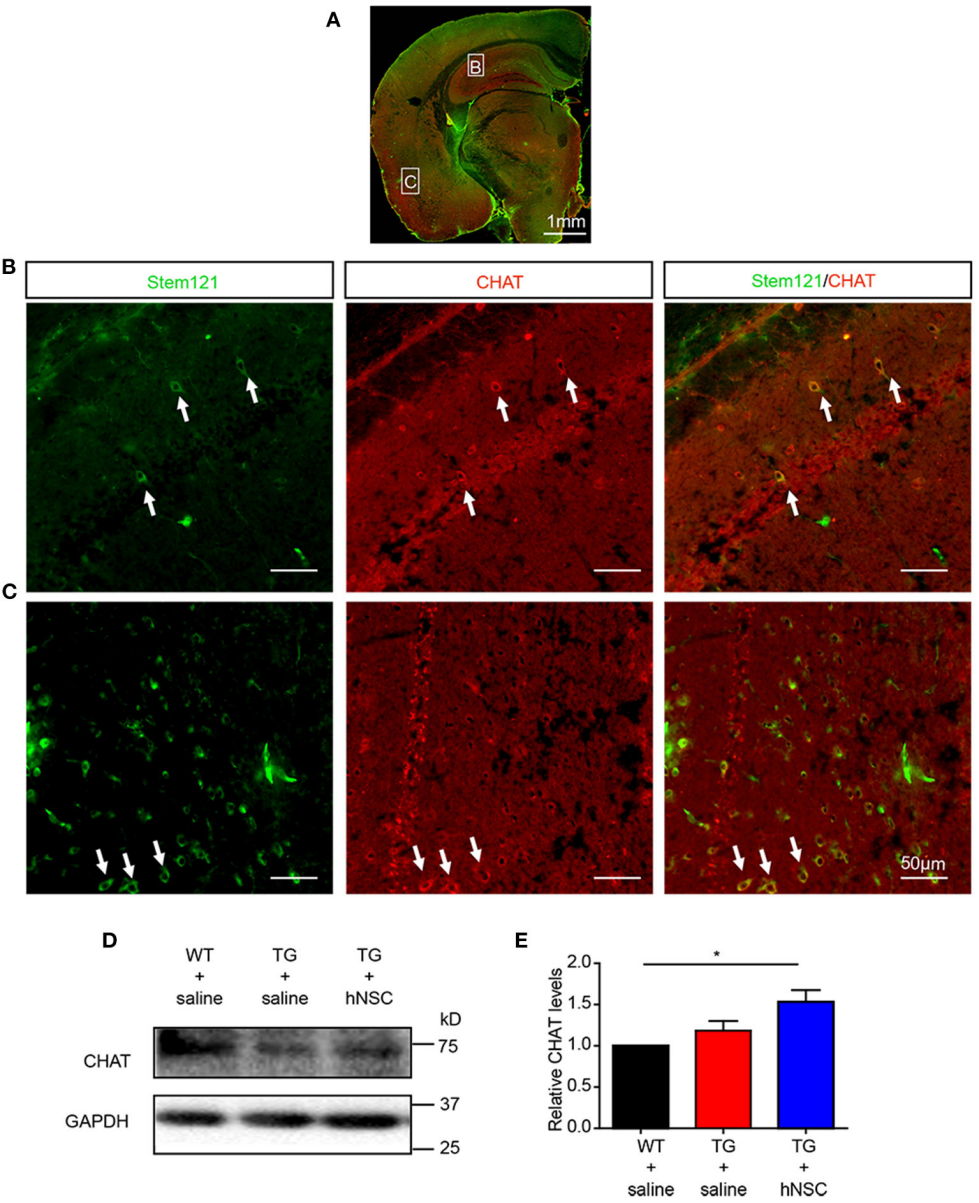
Transplanted hNSCs differentiate into cholinergic neurons in the brains of the host APP/PS1 transgenic mice. **(A–C)** The coronal sections of the host mice were coimmunostained with anti-stem121 and anti-CHAT antibodies. **(A)** The overall view of the fluorescence staining. **(B,C)** The enlarged images in the corresponding white square boxes shown in **(A)**. Hippocampus **(B)** and ventral cortex **(C)**. The white arrows indicate the hNSCs which were differentiated into cholinergic neurons. Scale bars: 1 mm **(A)**, 50 μm **(B,C)**. **(D,E)** The levels of CHAT in the hippocampus of host mice analyzed with Western blot. Relative levels of CHAT were quantified with those in WT mice were normalized to 1.0. Data are presented as mean ± SEM. *n* = 6 mice/group. *p < 0.05, One-way ANOVA **(E)**.

Cholinergic neuronal dysfunction and degeneration is one of the early pathological events in AD, which contributes to cognitive deficits (Hampel et al., 2018; Richter et al., 2018). To further investigate the differentiation capability of the intranasally transplanted hNSCs, we investigated whether these transplanted hNSCs could differentiate into cholinergic neurons, whose dysfunction contributes to AD pathogenesis (Hampel et al., 2018; Richter et al., 2018). Excitingly, quite a large number of cholinergic neurons differentiated from transplanted hNSCs were observed in the cortex, hippocampus, pons, and other regions (Figures 4A–C). Consistent with this result, the protein level of cholinergic transferase (CHAT), which is responsible for the synthesis of acetylcholine, increased in the hippocampus of APP/PS1 mice following hNSCs delivery (Figures 4D,E). These data indicate that intranasally transplanted hNSCs differentiate to functional cholinergic neurons in APP/PS1 mice.

### Intranasal Transplantation of hNSCs Attenuates Aβ Accumulation in APP/PS1 Mice by Promoting Its Clearance

**Figure 5:**
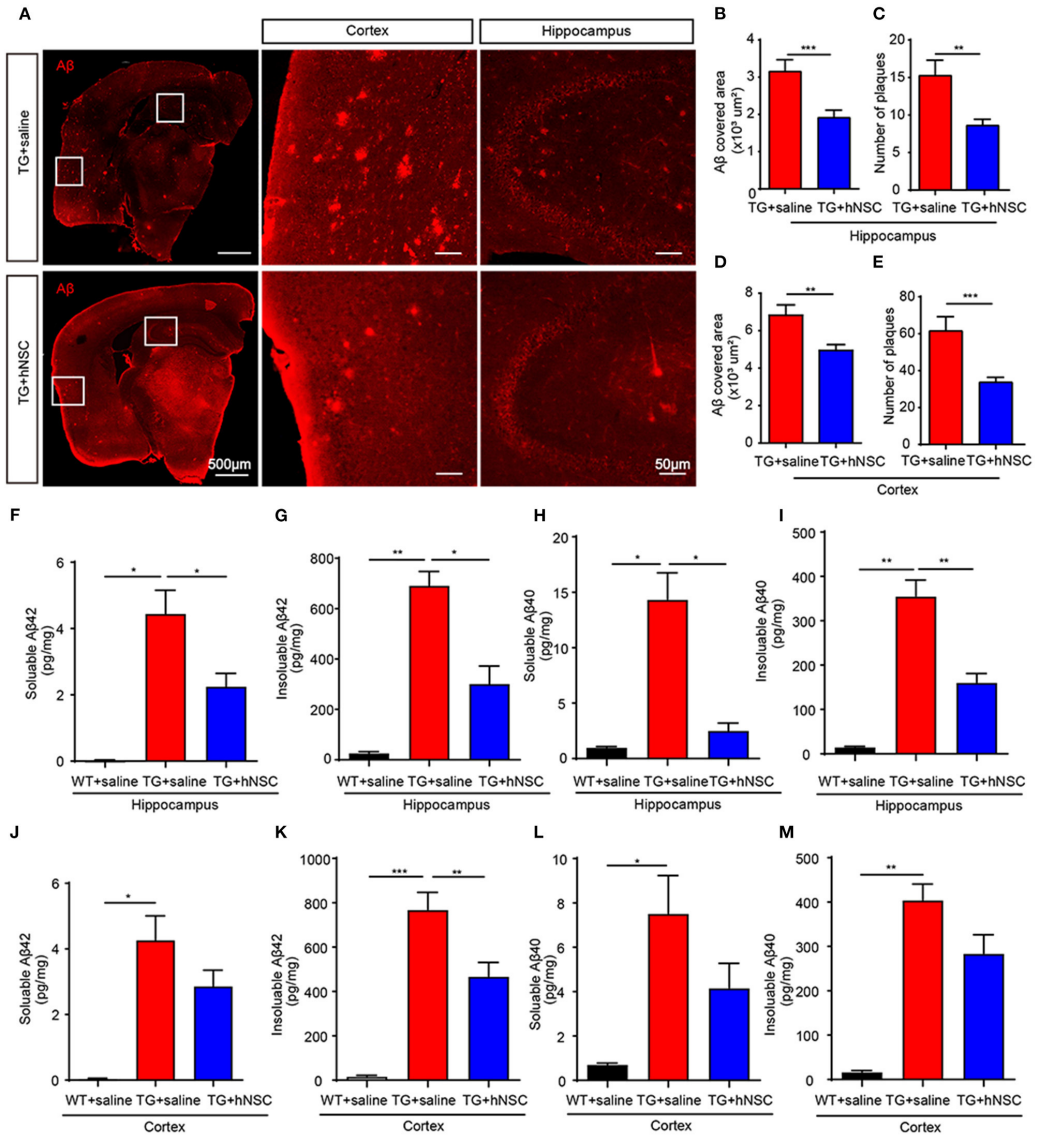
Intranasal transplantation of hNSCs reduces Aβ accumulation in the brains of APP/PS1 mice. **(A–E)** The coronal sections of host and control APP/PS1 mice were stained with an antibody against Aβ (6E10). Representative images in the hippocampus and cortex are shown. Scale bars: 100 and 50 μm in the images with higher magnification. The size of Aβ plaques in the hippocampus **(B)** and cortex **(D)**. The numbers of Aβ plaques in the hippocampus **(C)** and cortex **(E)**. **(F–M)** ELISA analysis of the levels of soluble and insoluble Aβ42 **(F,G,J,K)** and Aβ40 **(H,I,L,M)** in the cortex and hippocampus. Data are presented as mean ± SEM. *n* = 20–40 slices from 5 mice/group for Aβ plaques analysis; *n* = 5 mice/group for ELISA analysis. *p < 0.05; **p < 0.01; ***p < 0.01. Student *t*-test **(B–E)**. One-way ANOVA **(F–M)**.

Aβ is one of the key initiating factors of AD pathogenesis. Accumulation of Aβ results in loss of synapses, neuroinflammation, and ultimately cognitive deficits (Musiek and Holtzman, 2015). Thus, we investigated the function of hNSCs on Aβ accumulation. Aβ staining was performed using an antibody against Aβ in mouse coronal sections. The numbers and size of Aβ plaques were quantified in the brain sections from APP/PS1 mice transplanted with hNSCs and the age-matched APP/PS1 controls. The results showed that both the size and the numbers of Aβ plaques in the hippocampus (Figures 5A–C) and cortex (Figures 5A,D,E) of hNSCs-transplanted APP/PS1 mice were decreased compared with those in control APP/PS1 mice. Consistent with these results, ELISA analysis showed that the levels of both soluble and insoluble Aβ42 and Aβ40 in the hippocampus of hNSCs-transplanted APP/PS1 mice were also decreased (Figures 5F–I). The cortical insoluble Aβ42 also exhibited reduced levels upon hNSCs transplantation (Figure 5K). Whereas, soluble Aβ42 and Aβ40, together with insoluble Aβ40 in the cortex of hNSCs-transplanted APP/PS1 mice showed a tendency to decrease, although this did not reach a statistic difference (Figures 5J,L,M). These results indicate that repetitive intranasal transplantation of hNSCs decreases Aβ accumulation in the brains of APP/PS1 mice.

**Figure 6:**
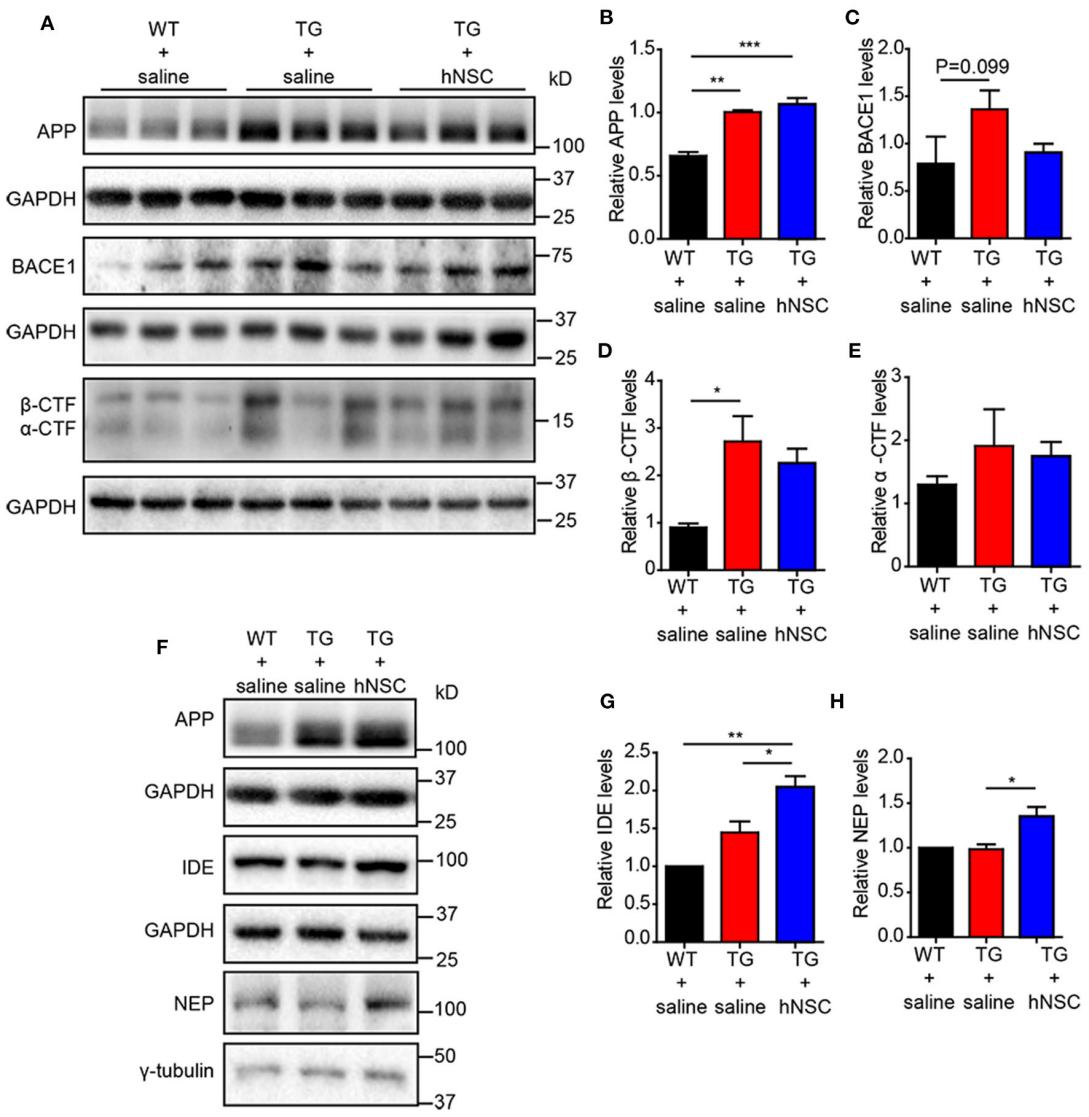
Intranasal transplantation of hNSCs increases Aβ-degrading enzymes without altering the cleavage of APP. Western blot analysis of the levels of full-length APP **(A,B)**, BACE1 **(A,C)**, β-CTF **(A,D)**, α-CTF **(A,E)**, IDE **(F,G)**, and NEP **(F,H)** in the hippocampus of host mice. **(B–E,G,H)** The relative levels of the above proteins. The level of IDE and NEP were quantified with those in WT mice being normalized to 1.0 **(G,H)**. Data are presented as mean ± SEM. *n* = 6 mice/group. *p < 0.05; ***p < 0.01. One-way ANOVA.

Reduced Aβ levels could be caused by insufficient production capacity or increased clearance. Aβ is produced by the cleavage of amyloid precursor protein (APP) by β- and γ-secretase sequentially. The cleavage of APP by α/β-secretase generates a carboxy terminal fragment called α-/β-CTFs. Thus, the levels of α-/β-CTFs could reflect the cleavage of APP by α-/β-secretase. However, neither α-/β-CTFs nor full-length APP exhibited altered levels upon hNSCs transplantation. The levels of β**-**site APP-cleaving enzyme 1 (BACE1) showed a trend of reduction in the hippocampus of hNSCs-transplanted APP/PS1 mice in comparison to those in control APP/PS1 mice (Figures 6A–E). We thus further investigated the possibility that hNSCs transplantation affects the clearance of Aβ by examining the levels of IDE and NEP, two key enzymes in charge of Aβ degradation (Miners et al., 2010; Kurochkin et al., 2018). The results demonstrated both IDE and NEP levels were increased in the hippocampus of hNSCs transplanted mice compared to age-matched control APP/PS1 mice (Figures 6F–H). Thus, these results indicate that repetitive intranasal transplantation promotes Aβ clearance by enhancing Aβ degradation.

### hNSCs Inhibit Neuroinflammation in APP/PS1 Mice

**Figure 7:**
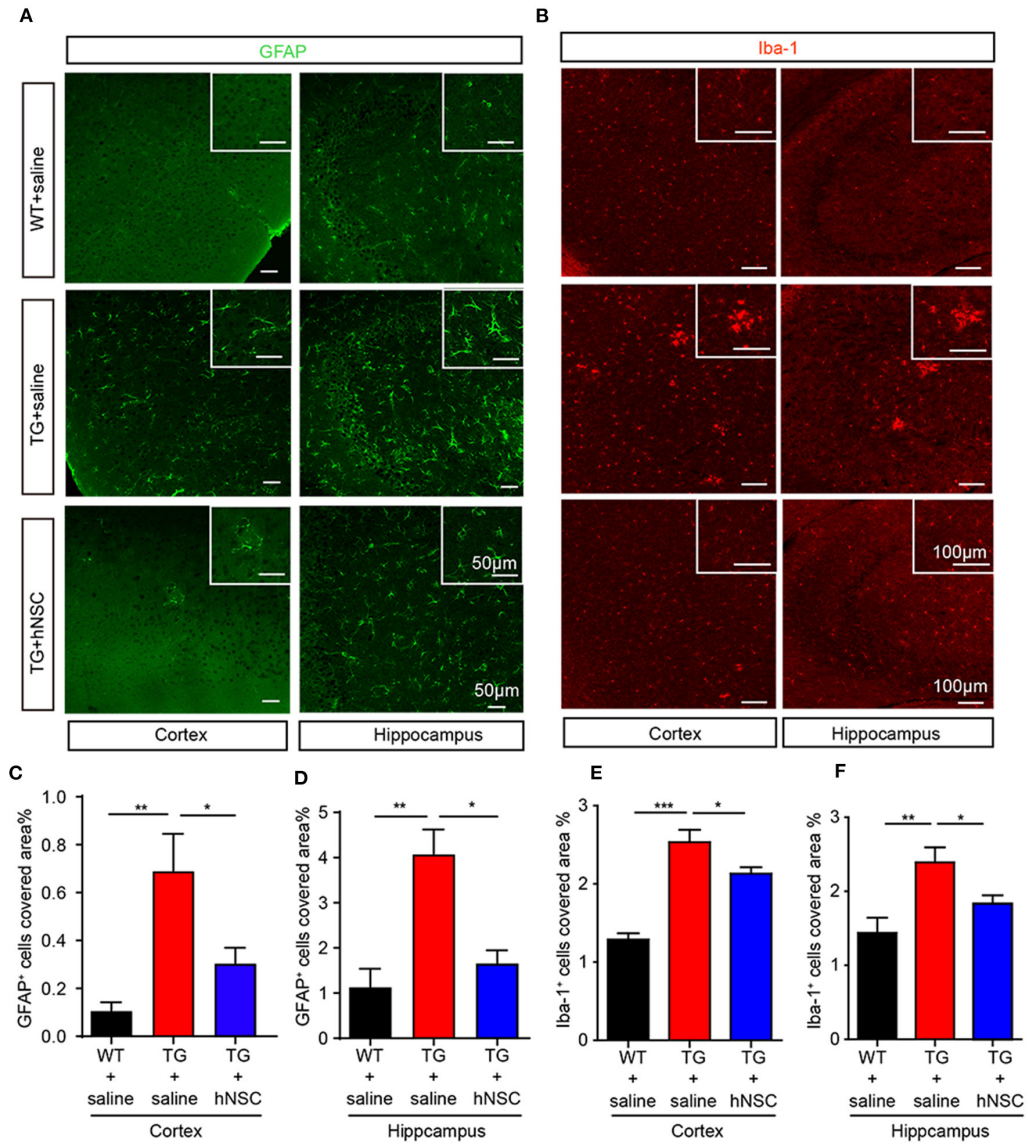
Intranasal transplantation of hNSCs attenuates neuroinflammation in the brains of APP/PS1 mice. The coronal hippocampal and cortical section were coimmunostained for GFAP **(A)** or Iba-1 **(B)**. Scale bars: 50 μm **(A)** and 100 μm **(B)**. The percentage of the area of astrocytes **(C,D)** or Iba-1 **(E,F)** occupied in the total area in the cortex and hippocampus was quantified. Data are presented as mean ± SEM. *n* = 19–37 slices from 6 mice/group. *p < 0.05; **p < 0.01; ***p < 0.01. One-way ANOVA.

Neuroinflammation is widely reported as one of the mechanisms of Alzheimer's disease pathology (Calsolaro and Edison, 2016). Excessive neuroinflammation leads to synaptic degeneration and memory loss. We thus further examined whether intranasally transplanted hNSCs attenuate neuroinflammation in the brains of APP/PS1 mice by quantifying the density of microglia and astrocytes, the glial cells responsible in initiating neuroinflammation in the brain. The coronal sections of transgenic mice were immunostained with antibodies of Iba1 (marker of microglia) and GFAP (marker of astrocytes), respectively. As expected, saline-injected APP/PS1 mice exhibited a significantly increased density of astrocytes (Figures 7A,C,D) and microglia (Figures 7B,E,F) in both the hippocampus and cortex compared with WT mice. Whereas, hNSCs transplantation reduced the density of astrocytes (Figures 7A,C,D) and microglia (Figures 7B,E,F) to a level comparable to that of WT mice. These findings demonstrate that repetitive intranasal transplantation of hNSCs rescues neuroinflammation in the brains of APP/PS1 mice.

### Intranasal Transplantation of hNSCs Enhances Endogenous Neurogenesis, While Ameliorating Pericytic and Synaptic Loss in the Hippocampus of APP/PS1 Mice

**Figure 8:**
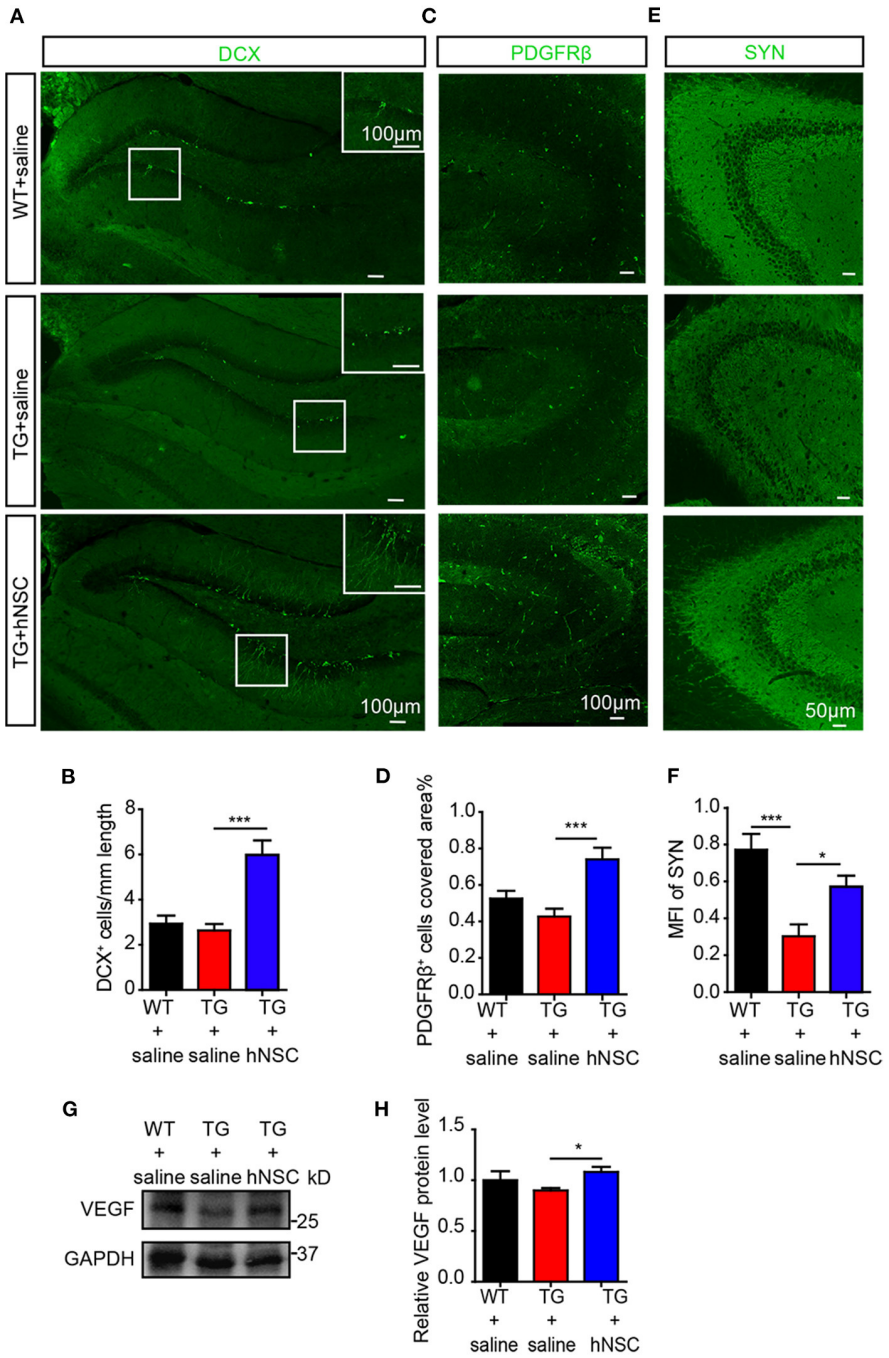
Intranasal transplantation of hNSCs enhances hippocampal neurogenesis, VEGF level, and synaptic density of APP/PS1 mice. The coronal hippocampal sections were immunostained for either DCX **(A)** or synaptophysin (**E**, SYN) and PDGFRβ **(C)**. The numbers of DCX^+^ cells **(B)**, PDGFRβ^+^ cells **(D)**, and the mean fluorescence intensity (MFI) of SYN **(F)** were quantified. Scale bars: 100 μm **(A,C)**, 50 μm **(E)**. The level of VEGF in mice hippocampus were analyzed by Western blotting **(G)** and quantified **(H)**. Data are presented as mean ± SEM. *n* = 21–28 slices from 4 to 6 mice/group for SYN stanning; *n* = 13–31 slices for DCX stanning and *n* = 6–17 slices for PDGFRβ stanning from 3 to 5 mice/group. *p < 0.05; **p < 0.01; ***p < 0.001. One-way ANOVA.

AHN displays an obvious potential for structural plasticity, which is closely related to cognition (Trinchero et al., 2017). The declined AHN is an early event in AD, even prior to the occurrence of Aβ deposition (Tobin et al., 2019). We next sought to evaluate whether intranasal transplantation of hNSCs would affect AHN of APP/PS1 transgenic mice. DCX, a marker of immature newborn neurons, were detected exclusively in the sub-granular layer of DG. Strikingly, the numbers of DCX^+^ cells increased by 2–3 times in the DG of hNSC-transplanted APP/PS1 mice compared to those in saline-treated APP/PS1 mice. Moreover, the DCX^+^ cells in hNSCs-transplanted APP/PS1 DG harbored more and longer dendritic branches that extended well into the molecular layer, compared to those in saline-treated APP/PS1 DG (Figures 8A,B). Thus, these results indicate that intranasal transplantation of hNSCs enhances AHN of APP/PS1 mice.

Pericytes degeneration in AD has been reported both in post-mortem brains in human and in AD mouse models (Sagare et al., 2013; Halliday et al., 2016). Loss of pericytes contributes to AD pathogenesis by multiple mechanisms including regulating Aβ clearance by phagocytosis and transportation of Aβ out of CNS through the BBB, maintaining BBB permeability, supplying oxygen, and metabolites in collaboration with the endothelial cells (Rustenhoven et al., 2017; Ma et al., 2018; Nortley et al., 2019). Pericytes also modulate neurogenesis and angiogenesis (Motegi et al., 2011; Farahani et al., 2019), which are correlated with cognition as well. We thus examined the role of transplanted hNSCs on pericytic degeneration by immunostaining PDGFRβ, a specific marker of pericytes in the coronal section of the mouse brain. Our data showed that the density of pericytes in the hippocampus of APP/PS1 mice increased significantly upon hNSCs transplantation (Figures 8C,D).

Synapses are essential for cognitive function, whose loss is closely related to the cognitive impairment of AD (Robinson et al., 2014; Scheff et al., 2014). We wondered whether hNSCs ameliorates synaptic loss. We assessed synaptic density in the hippocampal CA3 region, where it is rich in synapses, by immunostaining SYN, a marker for presynaptic proteins. Mean fluorescence intensity of SYN^+^ signals in the CA3 region of APP/PS1 mice was increased following hNSCs transplantation (Figures 8E,F). These data provide evidence that intranasal transplantation of hNSCs ameliorates synaptic loss in AD.

Thus, repetitive intranasal transplantation of hNSCs enhanced neurogenesis, preventing the loss of pericytes and synapses in the brains of APP/PS1 mice. To further explore the mechanisms, we examined the levels of growth factors such as brain derived neurotrophic factor (BDNF) and nerve growth factor (NGF) and neurotrophin such as neurotrophin 3 (NTF3), which play essential roles in the above processes. However, none of them exhibited different levels in the brains of hNSCs-transplanted and control APP/PS1 mice (Supplementary Figure 1). In contrast, the level of vascular endothelial growth factor (VEGF), which harbors neuroprotective function, enhancing neurogenesis and angiogenesis (Sun et al., 2003; Melincovici et al., 2018), was significantly elevated in the hippocampus of APP/PS1 mice which were transplanted with hNSCs (Figures 8G,H), suggesting a potential mechanism underlying the beneficial effects of intranasal transplantation of hNSCs.

### Intranasally Transplanted hNSCs Ameliorates Cognitive Deficits of APP/PS1 Mice

**Figure 9:**
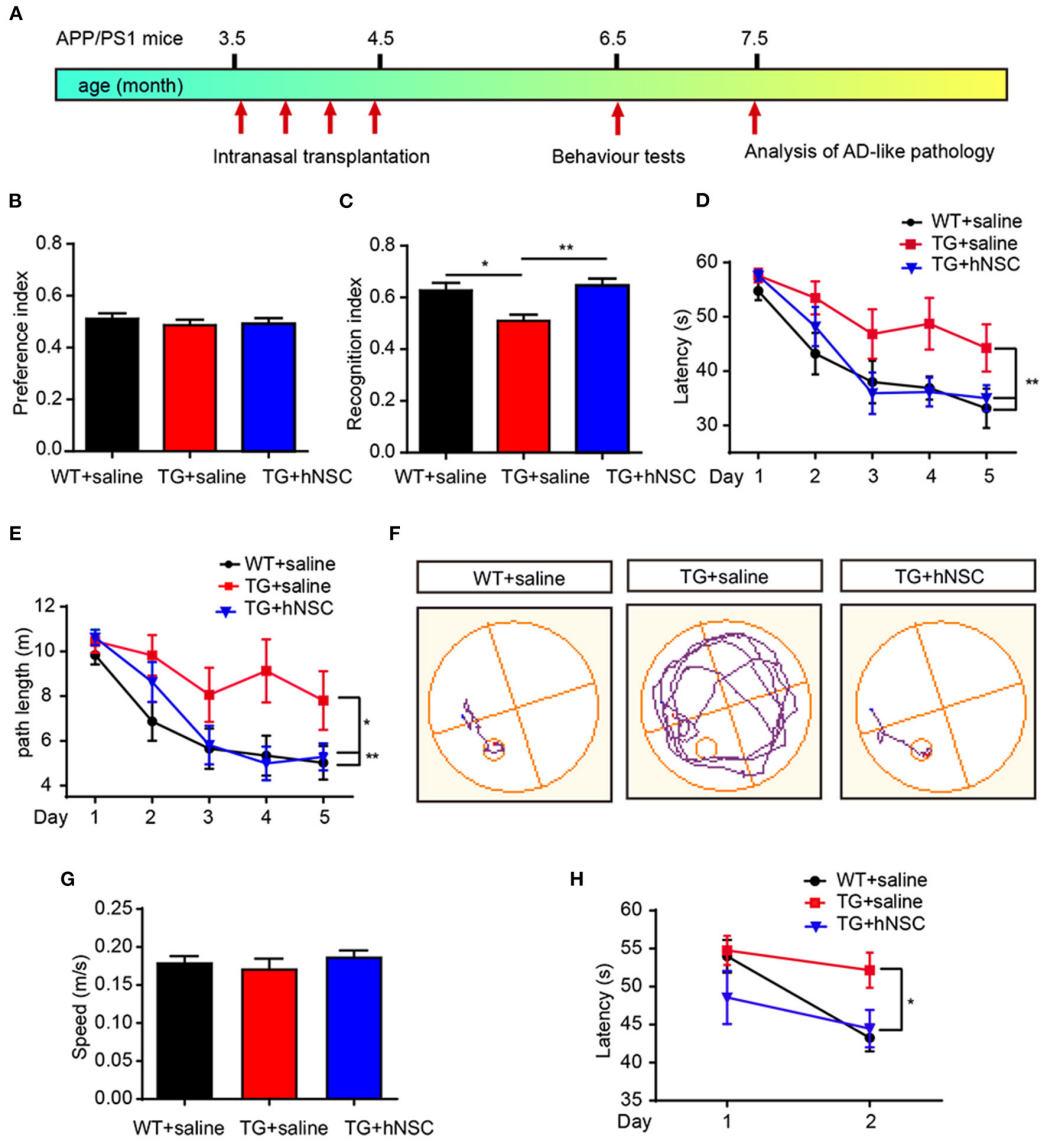
Intranasal transplantation of hNSCs rescues cognitive deficits of APP/PS1 mice. **(A)** Schematic diagram of experimental design. 3.5-month-old APP/PS1 mice were transplanted with hNSCs by intranasal administration four times with a frequency of once a week, and were subjected to behavior test at 6.5 months. **(B,C)** Novel object recognition test. Location preference **(B)** and recognition index **(C)**. **(D–H)** Morris water maze test. Escape latencies **(D)**, swimming path length **(E)**, representative track plots **(F)**, swimming speed **(G)**; the escape latencies after target platform were switched to the opposite quadrant **(H)**. Data are presented as mean ± SEM. *n* = 9–16 mice/group. *p < 0.05; **p < 0.01. One-way ANOVA **(B,C,G)**, Two-way ANOVA **(D,E,H)**.

Cognitive deficit is the main feature of AD pathology. We next examined the effect of intranasal transplantation of hNSCs on the cognition of APP/PS1 transgenic mice. hNSCs were transplanted intranasally to 3.5-month-old APP/PS1 transgenic mice a total of four times at a frequency of once a week, and a behavioral assessment was performed at 6.5 months when the APP/PS1 mice started to exhibit cognitive deficits (Figure 9A). Age-matched APP/PS1 and WT mice who received saline were used as the controls. The mice were subjected to novel object recognition (NOR) and Morris water maze (MWM) tests. In the NOR test, mice in all groups showed similar exploring time when objects were identical, suggesting they had no preference for object location (Figure 9B). When one of the objects was replaced with a new object, APP/PS1 mice failed to recognize the novel object as evidenced by the reduced recognition index. Whereas, hNSC-transplanted APP/PS1 mice exhibited an increased recognition index, even to a level comparable to WT mice (Figure 9C). These data indicate that intranasal transplantation of hNSCs rescues the impaired short-term memory of APP/PS1 mice. Mice were subsequently tested for spatial learning and memory by MWM. Control APP/PS1 mice showed impaired learning in locating the invisible platform as indicated by the longer escape latency and the longer swimming distance in the consecutive trials in comparison to WT mice. In contrast, hNSCs-transplanted APP/PS1 mice showed markedly improved learning capability, even to a level comparable with WT mice (Figures 9D–F). The above differences were not due to swimming speed, which exhibited identical levels among groups of mice (Figure 9G). To support this notion, these mice were given reversal learning tasks 2 weeks later for 2 consecutive days, where the platform was moved to the opposite quadrant. Nevertheless, only on the second day of training, hNSCs-transplanted APP/PS1 mice showed a significantly shorter escape latency compared to saline-treated APP/PS1 mice, that was comparable to WT mice (Figure 9H). Together, these results indicate that repetitive intranasal transplantation of hNSCs rescues deficits of learning and memory in APP/PS1 mice.

## Discussion

**Figure 10:**
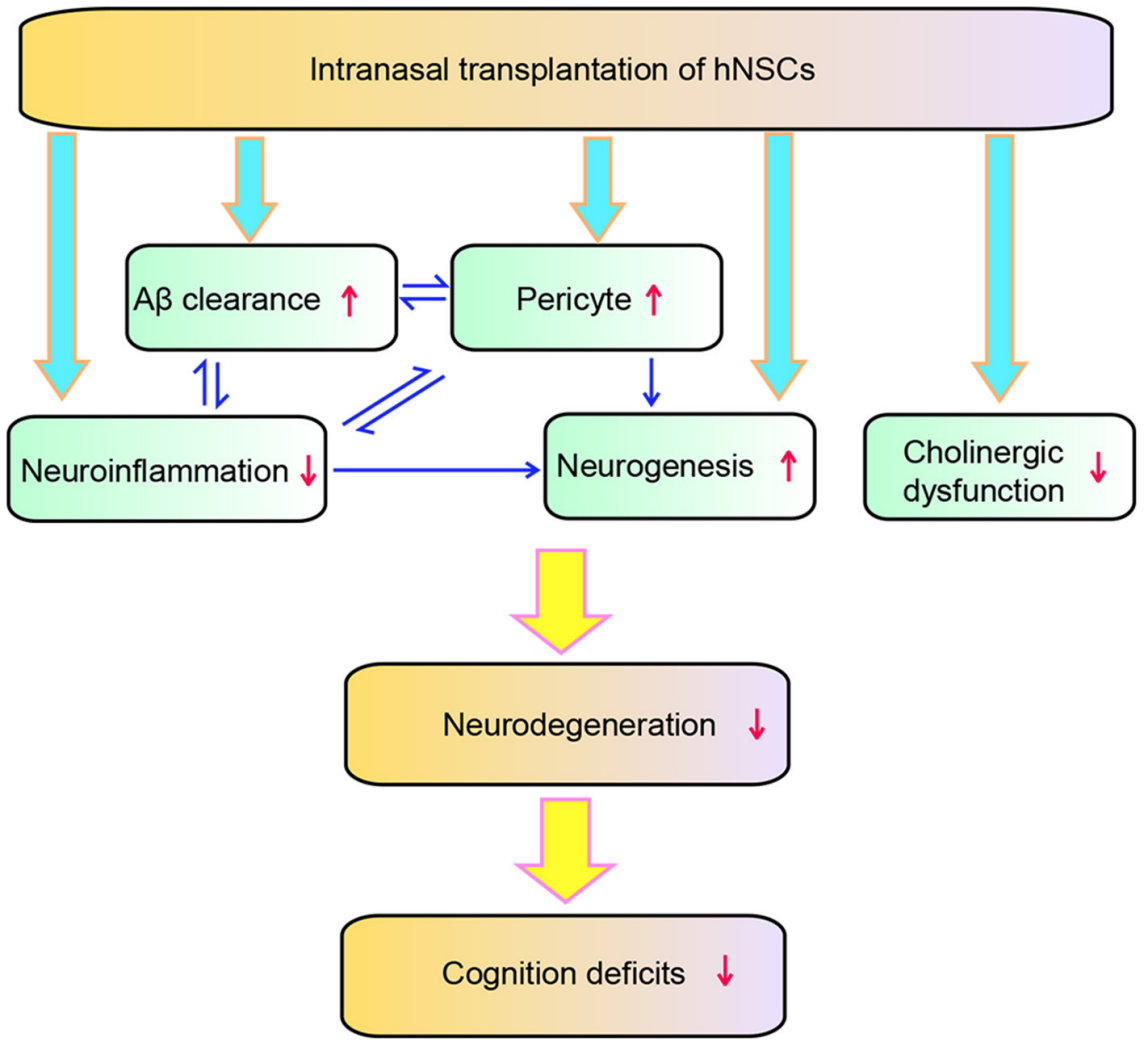
Schematic diagram of the effect of hNSCs on AD pathology. hNSCs play roles through multiple targets which interact with each other, and ultimately improves the degeneration and cognition deficits of AD.

In the present study, we demonstrated a therapeutic potential of repetitive intranasal transplantation of hNSCs in AD by using APP/PS1 transgenic mice. Our findings show that intranasal delivery of hNSCs, an invasive approach which allows repetitive administration of hNSCs, attenuates Aβ accumulation, neuroinflammation, pericytic and synaptic degeneration, and eventually rescuing the cognitive deficits of APP/PS1 mice. The intranasally transplanted hNSCs exhibited wide distribution in the host brains and neuronal and glial differentiation. Mechanistically, intranasal transplantation of hNSCs enhanced Aβ clearance as evidenced by increased expression of Aβ degraded enzymes including IDE and NEP. Intranasal transplantation of hNSCs attenuated pericytic and synaptic degeneration and neuroinflammation, the key pathological events of AD, while enhancing endogenous neurogenesis. In addition, intranasal transplantation of hNSCs also ameliorated cholinergic dysfunction in the brains of APP/PS1 mice as a high amount of transplanted hNSCs differentiated into cholinergic neurons. Therefore, intranasal transplantation of hNSCs attenuated AD pathology and cognitive deficits of the AD model mice via multiple mechanisms (Figure 10).

One of the limitations of stem cell transplantation in the therapy of neurological disorders is the delivery approach. So far, intracranial injection and intrathecal injection are the most common ways to delivery stem cells to the brain in the preclinical study and clinical study of neurological disorder therapy. However, some neurological disorders, such as neurodegenerative disorders, have long-term courses. And most patients are in old age which makes them more susceptible to infections and other risks brought on by surgery. Moreover, the invasive delivery approach limits the repetitive delivery of stem cells. Many studies have observed that the intranasal delivery of either small molecular substances or macromolecular proteins enables them to enter the CNS by passing the BBB (Banks, 2012). Stem cells have been observed to be capable of entering the brain when being delivered intranasally as well (Jiang et al., 2011; Galeano et al., 2018). We herein have observed that hNSCs which were administrated intranasally distribute well in various brain regions including the dorsal and ventral cortex, hippocampus, thalamus, olfactory bulbs, and cerebellum, all of which exhibit Aβ deposition (Braak and Braak, 1991; Cole et al., 1993). The intranasally transplanted hNSCs attenuated AD-like pathology in AD model mice. Thus, our present data indicate that intranasal administration is an alternative delivery approach for stem cell therapy in AD, which will not compromise the beneficial effects of stem cells.

We herein observed that intranasal transplantation of hNSC reduced Aβ accumulation, one of the key initiators of AD pathogenesis. The accumulation of Aβ resulted from an imbalance of the production and clearance of Aβ. We thus further examined these two processes. Intranasal transplantation of hNSCs seemed to have no effect on the production of Aβ, as evidenced by the levels of full-length APP and β-CTFs which remained unchanged in hNSC-transplanted APP/PS1 mice, despite that BACE1 showed a trend of reduction. Surprisingly, the levels of NEP and IDE, two enzymes degrading Aβ, were elevated by intranasal transplantation of hNSCs. These results indicate that intranasal transplantation of hNSCs promoted Aβ clearance by enhancing the expression of Aβ-degrading enzymes. However, in addition to the proteolytic clearance of Aβ, glia-mediated phagocytosis also plays an essential role in Aβ clearance. hNSC modulates the phagocytotic capability of microglia when being cocultured with microglia (McGinley et al., 2018). In this context, we observed that the density of astrocytes and microglia, two types of glia involved in the clearance of Aβ following phagocytosis, was also decreased by intranasal transplantation of hNSCs. However, we cannot rule out the possibility that the decreased neuroinflammation may be directly caused by the reduction of Aβ. Moreover, intranasal transplanted hNSCs prevented the loss of pericytes in APP/PS1 mouse brains, which regulated Aβ clearance via transportation of Aβ through the BBB out of the CNS (Ma et al., 2018). Thus, the decreased levels of Aβ upon intranasal transplantation of hNSCs may be due to both the upregulated expression of NEP and IDE and the enhanced glia- and pericyte-mediated phagocytosis.

Consistent with the data obtained *in vitro*, the intranasally transplanted hNSCs differentiated more neurons rather than glial cells. Moreover, a proportion of transplanted hNSCs differentiated into cholinergic neurons, which exhibited degeneration and contributed to the early attention and memory dysfunction in AD (Hampel et al., 2018). The transplanted hNSCs-derived cholinergic neurons *may be f* unctional as the reduced levels of CHAT have been restored upon transplantation of hNSCs. Thus, the improved cognition by transplantation of hNSCs may be also contributed to by attenuated cholinergic dysfunction. This result is also consistent with previous studies which show that intracranial transplantation of either ESC-derived cholinergic neurons or CHAT-overexpressing NSCs alleviates the cognitive deficits in AD model mice (Gu et al., 2015; Yue et al., 2015; Park et al., 2020).

AHN plays essential roles in the maintenance of cognition. Declined AHN has been found to be altered in AD patients (Moreno-Jimenez et al., 2019). Impaired AHN occurs even prior to the onset of classical AD pathology in AD model mice, e.g., Aβ deposition (Mu and Gage, 2011). The impaired AHN causes neuronal and synaptic degeneration, and exacerbated cognitive impairment in AD model mice (Choi et al., 2018). Consistent with this study, we herein observed that intranasal transplantation of hNSCs enhanced AHN, which is concomitant with attenuated synaptic degeneration. We surprisingly observed that intranasal transplantation rescued the loss of pericytes in the brains of AD model mice, which plays essential roles in AD pathogenesis by regulating Aβ clearance, neuroinflammation, BBB permeability, oxygen and metabolites supply, angiogenesis, and neurogenesis (Motegi et al., 2011; Rustenhoven et al., 2017; Ma et al., 2018; Farahani et al., 2019; Nortley et al., 2019). Thus, the beneficial effects brought on by intranasal transplanted hNSCs may also be contributed to by the rescued pericyte loss. The hNSCs could secrete a variety of neurotrophic factors, including BDNF, FGF2, VEGF, NTF3, and GDNF (Lee et al., 2015), which offers beneficial effects to the host upon transplantation via a paracrine mechanism (Blurton-Jones et al., 2009; Sun and Ma, 2013). It is worth noting that, we failed to observe altered levels of growth factors such as BDNF and NGF and neurotrophin such as NTF3 by intranasal transplantation. In contrast, VEGF levels were increased by intranasal transplantation of hNSCs. VEGF exhibits protective functions which prevents degeneration of neurons and pericytes, in addition to enhancing neurogenesis and angiogenesis (Melincovici et al., 2018). Moreover, VEGF enhances the proliferation of pericytes when they are cocultured with endothelial cells (Bowers et al., 2020), which may contribute to the increased density of pericytes by transplantation of hNSCs as well. In addition to hNSCs, VEGF can be secreted by endogenous neurons, pericytes, and newborn neurons. Thus, the increased VEGF may be ascribed to these cells as well. However, it is worth noting that the failure of detecting changes of other neurotrophic factors could also be due to the fact that these factors decreased their levels with the differentiation of transplanted hNSCs. Thus, it is possible a paracrine mechanism is also involved in the beneficial effects observed in this study. Thus, the enhanced densities of newborn neurons, synapses, and pericytes by transplanted hNSCs may ne due to the elevated levels of VEGF. These multiple beneficial effects brought on by hNSCs attenuates the hostile environment, further enhancing the efficacy of increased AHN in cognition as that enhancing AHN alone without changing the hostile environment fails to improve cognition in AD model mice (Choi et al., 2018).

In conclusion, our study suggests that repetitive intranasal transplantation of hNSCs exerts beneficial effects via multiple mechanisms such as attenuating Aβ accumulation, neuroinflammation, cholinergic and loss of pericytes, and enhancing AHH. These multiple mechanisms work coordinately by interacting with each other, eventually attenuating synaptic degeneration and improving cognition in AD (Figure 10).

## Data Availability Statement

The raw data supporting the conclusions of this article will be made available by the authors, without undue reservation.

## Ethics Statement

The animal study was reviewed and approved by Ethics Committee of Soochow University.

## Author Contributions

M-HL, W-LJ, HC, Y-YS, YS, Y-NH, and B-XL performed the experiments and analyzed the data. J-WZ isolated and cultured the hNSCs. FW and D-EX assisted in designing the experiments. Q-HM and M-HL designed the project and wrote the manuscript. J-wW and C-FL revised the manuscript. All authors contributed to the article and approved the submitted version.

## Conflict of Interest

J-WZ is employed by Angecon Biotechnology Co. Ltd. The remaining authors declare that the research was conducted in the absence of any commercial or financial relationships that could be construed as a potential conflict of interest. The authors declare that this study received funding from Angecon Biotechnology Co. Ltd. The funder had the following involvement with the study: They provided hNSCs and are responsible for identifying the characteristics, purity and differentiation of hNSCs in vitro.

## Supplementary Material

Supplementary Figure 1 The levels of growth factors such as BDNF and NGF, and NTF3. The levels of BDNF **(A)**, NGF **(B)**, and NTF3 **(C)** in the cortex of animal brains from each group were measured by ELISA. *n* = 6 mice/group.

## Abbreviations

Aβ: β-amyloid
AD: Alzheimer's disease
AHN: adult hippocampal neurogenesis
APP: amyloid precursor protein
BACE1: beta-site APP-cleaving enzyme 1
BBB: blood brain barrier
BDNF: brain-derived neurotrophic factor
CHAT: cholinergic transferase
CNS: central nervous system
DCX: doublecorxin
DG: dentate gyrus
ELISA: enzyme linked immunosorbent assay
hNSCs: human neural stem cells
IDE: insulin-degrading enzyme
MWM: Morris water maze
NEP: neprilysin
NGF: nerve growth factor
NOR: novel object recognition
NTF3: neurotrophin 3
VEGF: vascular endothelial growth factor
SYN: synaptophysin.
