# Supplementary figures and images for: Intranasal Transplantation of Human Neural Stem Cells Ameliorates Alzheimer's Disease-Like Pathology in a Mouse Model
Source: Front Aging Neurosci. 2021 Mar 10;13:650103. doi: 10.3389/fnagi.2021.650103 (PMC7987677; doi:10.3389/fnagi.2021.650103)

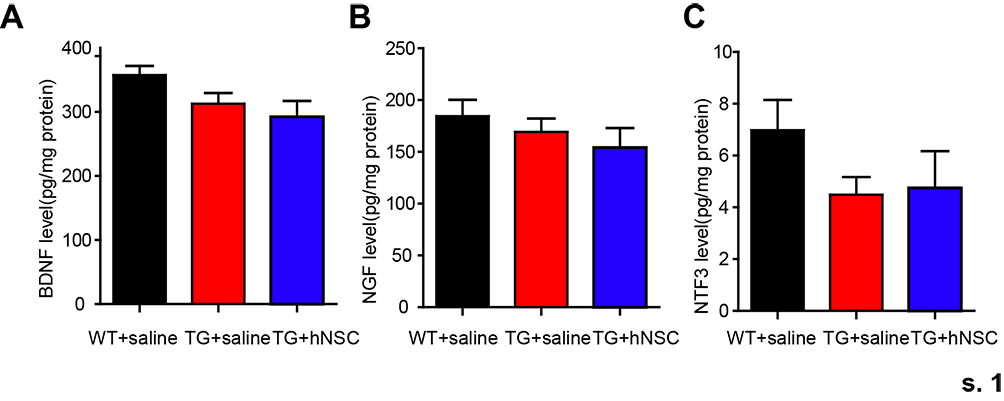

Supplement: Supplementary Figure 1 — The levels of growth factors such as BDNF and NGF, and NTF3. The levels of BDNF (A), NGF (B), and NTF3 (C) in the cortex of animal brains from each group were measured by ELISA. n = 6 mice/group. [file Image_1.TIF]
